# Editorial: Targeting Cardiac Proteotoxicity

**DOI:** 10.3389/fphys.2021.669356

**Published:** 2021-03-25

**Authors:** Mark J. Ranek, Md. Shenuarin Bhuiyan, Xuejun Wang

**Affiliations:** ^1^Department of Medicine, Division of Cardiology, The Johns Hopkins Medical Institutions, Baltimore, MD, United States; ^2^Department of Pharmacology and Molecular Sciences, The Johns Hopkins University School of Medicine, Baltimore, MD, United States; ^3^Department of Pathology and Translational Pathobiology, Louisiana State University Health Sciences Center, Shreveport, LA, United States; ^4^Department of Molecular and Cellular Physiology, Louisiana State University Health Sciences Center, Shreveport, LA, United States; ^5^Division of Basic Biomedical Sciences, The University of South Dakota Sanford School of Medicine, Vermillion, SD, United States

**Keywords:** autophagy, proteasome, mitochondria, heart disease, chaperone, small heat shock protein, protein quality control, increased proteotoxic stress

Misfolded proteins not only lose their normal functions but also perturb other proteins, organelles, and cellular processes, causing cell malfunction and cell death (Wang et al., 2008). Proteotoxicity refers to all damaging effects exerted by misfolded proteins in the cell (Sandri and Robbins, 2014). To sense and minimize the level and toxicity of misfolded proteins, the cell has developed multi-layered protein quality control (PQC) mechanisms. PQC is performed by the intricate collaboration between molecular chaperones and targeted protein degradation; the latter involves primarily the ubiquitin-proteasome system (UPS) and autophagy (Wang et al., 2008). Both increased production of misfolded proteins and PQC impairment can lead to an increased proteotoxic stress state (IPTS), which can result from genetic mutations, environmental stressors, aging process, and even chemotherapies (Wang and Wang, 2020).

Over the last two decades, it is increasingly evident that IPTS contributes to the genesis of a large subset of heart failure (HF) (Willis and Patterson, 2013, Wang and Wang, 2020). During cardiac IPTS, the accumulation of misfolded proteins and resultant aberrant protein aggregation further impair PQC, compromise the integrity of contractile apparatus and organelles (e.g., mitochondria), and cause cardiomyocyte death, eventually leading to HF (Wang et al., 2008). Recent advances in the molecular mechanisms regulating proteasome- and lysosome-mediated protein degradation promise new strategies to prevent or more effectively treat HF via targeting cardiac proteotoxicity. This is exciting as there are no current HF therapies aimed at targeting cardiac proteotoxicity. The present Research Topic, *Targeting Cardiac Proteotoxicity*, not only reports new work of cardiac IPTS but also discusses the current understanding, knowledge gap, potential molecular mechanisms and targets, and the obstacles to broad clinical implementation.

Autophagy is pivotal to both PQC and organelle quality control. Hence, suppressing autophagy is generally detrimental and, conversely, enhancing autophagy protects against IPTS (Wang and Cui, 2017). This is well-reflected in this *Research Topic*. LMP10 (β2i) is a proteolytic subunit of the immunoproteasome, which is the primary form of proteasomes in immune cells and an inducible form in non-immune cells (Aki et al., 1994). Yan et al. reveal that LMP10 deficiency attenuates maladaptive cardiac remodeling induced by angiotensin-II infusion in mice, perhaps through enhanced autophagic degradation of insulin growth factor receptor 1 and glycoprotein 130. This study supports the notion that proteasome malfunction activates autophagy (Pan et al., 2020); however, a direct anti-inflammatory effect from inhibition of immunoproteasomes remains possible. Consistent with this possibility, Guo et al. show that shikonin, an anti-inflammatory compound extracted from natural herbs, protects the heart from sepsis-induced injury. Notably, shikonin was shown by others to inhibit proteasome activities in macrophages (Lu et al., 2011). Occasionally a normally protective factor may become detrimental when autophagy becomes impaired or insufficient in the cell (Wang and Cui, 2017). This is exemplified by the perplexing effect of nuclear factor erythroid factor 2-related factor (Nrf2) in cardiac disease, as reviewed by Zang et al.

Since late 2019, all of humanity has been afflicted by the COVID-19 pandemic caused by the severe acute respiratory syndrome coronavirus 2 (SARS-CoV-2). A timely review by Gupta and colleague summarizes what we have learned during the 12 months following the first report of COVID-19, particularly the interaction between SARS-CoV-2 and autophagy. They describe that once SARS-CoV-2 enters the host cell, it can be degraded via autophagy or highjack the replication machinery. After evading degradation in the lysosome SARS-CoV-2 can replicate in an autophagosome, thereby reducing the influence of lysosomes on COVID-19 pathogenesis, which helps explain why autophagosome-lysosome fusion inhibitors (e.g., hydroxychloroquine) failed to meet the promises made early in the COVID-19 pandemic.

Mitophagy removes damaged mitochondria to limit reactive oxygen species production, crucial to maintaining mitochondrial, thus cardiomyocyte health (Tong et al., 2020). Impaired mitophagy contributes to doxorubicin-mediated cardiotoxicity, where the development of cardiomyopathy limits the clinical use of this powerful anti-neoplastic drug. Xu et al. report that Luteolin, a natural product extracted from plants alleviates doxorubicin-induced cardiotoxicity via enhancing mitophagy, effects that were abrogated with concomitant treatment with a Drp1 inhibitor, Mdivi-1. Kobayashi et al. explore the interplay of mitochondrial fission and mitophagy in a hyperglycemic model. Cultured cardiomyocytes subjected to high glucose displayed a marked reduction in mitophagy and mitochondrial health. Both genetic and pharmacological interrogations unveil that increasing mitophagy flux protects against high glucose induced cardiomyocyte injury. These studies add new evidence to the cardioprotective potential of enhancing mitophagy. The latter is comprehensively reviewed by Alam et al. which describes the potential role of mitochondrial proteostasis in cardiac IPTS, provides new insights into the mechanisms regulating mitophagy, and highlights potential targets for enhancing mitochondrial proteostasis to treat cardiac disease.

The contractile apparatus is central to the mechanical function of cardiomyocytes and is thereby under constant stress (Chung et al., 2016). Islam et al. review systematically small heat shock proteins that chaperone cytoskeletal integrity and the turnover of sarcomeres in cardiomyocytes. They propose a provocative “sarcostat” concept, a PQC mechanism located within the sarcomere that oversees sarcomere proteostasis. The authors also discuss potential targets for therapeutic intervention to enhance sarcomeric PQC. Further testing of the sarcostat model will be interesting. Mutations in genes encoding sarcomeric proteins cause cardiomyopathy, where pathogenesis likely involves IPTS as sarcomeric proteins are the most abundant proteins in cardiomyocytes (Willis and Patterson, 2013). McNamara et al. uncover an intronic variant of *TNNT2* that leads to the development of hypertrophic cardiomyopathy in felines. Distal arthrogryposis (DA) is another disorder that can be caused by genetic mutations affecting striated muscle, characterized by joint contractures, with the heart often affected. Desai et al. summarize the different forms of DA, their clinical features, and associated genetic mutations. It will be interesting to investigate how would the DA-linked mutations affect cardiac PQC.

Recent advances in the regulation of the protein degradation pathways have dramatically expanded our understanding of cardiac PQC. Li et al. review the role of a ubiquitin-like protein NEDD8. The covalent attachment of NEDD8 to substrate proteins is known as neddylation, critical to cardiac development and found to be dysregulated in vascular disease, liver disease, obesity, and HF. Oeing et al. highlight the newly identified roles of protein kinase G (PKG) in cardiac PQC that provide exciting new mechanism for PKG-mediated cardioprotection unveiled since early 2000s. Activation of PKG improves PQC via both priming the proteasome and increasing autophagy. The ability to activate PKG with drugs already in clinical use elevates the interest surrounding PKG as a therapeutic target for cardiac proteotoxicity.

Emerging evidence suggests inter-organ communication in proteotoxicity (Liu et al., 2021). To this end, Evangelisti et al. comprehensively contrast IPTS pathophysiology between the central nervous and the cardiovascular systems and nicely highlight the shared pathological features of IPTS diseases in the heart and brain, calling for studying IPTS in the two systems in parallel. Intriguingly, augmenting protein kinase A- or PKG-mediated proteasome activation reduces both cardiac and neural proteotoxicity in animal models (Zhang et al., 2019, Wang and Wang, 2020).

As illustrated by this series, the cardiac PQC field is bourgeoning and recently has made significant and exciting advances, providing new therapeutic targets that can be potentially translated into the clinic in the near future. Meanwhile, our understating of the molecular mechanisms underlying cardiac proteotoxicity remains quite limited. Continuing effort in defining pathways that regulate chaperones, the UPS, and autophagy is warranted for discovering new nodal points and methods for therapeutic intervention while we facilitate the translation of some of the most promising strategies to the clinic.

## Author Contributions

MR writing the first draft and grammatical polishing of the editorial. MB editing the draft of the editorial. XW planning, editing, and finalizing of the editorial, as well as response to editors' report. All authors contributed to the article and approved the submitted version.

## Conflict of Interest

The authors declare that the research was conducted in the absence of any commercial or financial relationships that could be construed as a potential conflict of interest.
